# Predicting the evolution of social networks with life cycle events

**DOI:** 10.1007/s11116-015-9644-8

**Published:** 2015-09-01

**Authors:** Fariya Sharmeen, Theo Arentze, Harry Timmermans

**Affiliations:** Eindhoven University of Technology, P.O. Box 513, Vertigo 8.09, 5600 MB Eindhoven, The Netherlands

**Keywords:** Social network dynamics, Micro-simulation, Life-cycle events, Tie formation, Tie dynamics

## Abstract

This paper presents a model of social network evolution, to predict and simulate changes in social networks induced by lifecycle events. We argue that social networks change with lifecycle events, and we extend a model of friendship selection to incorporate these dynamics of personal social networks. The model uses theories of homophily and reciprocity and is formulated in a random utility maximization framework to predict the formation of social ties between individuals in the population. It is then extended to predict the evolution of social networks in response to life cycle events. The model is estimated using attribute data of a national sample and an event-based retrospective dataset collected in 2009 and 2011 respectively. Findings suggest that homophily has a strong effect on the formation of new ties. However, heterophily also plays a role in maintaining existing ties. Although the motivation of this research stems from incorporating social network dynamics in large-scale travel behaviour micro-simulation models, the research can be used in a variety of fields for similar purposes.

## Introduction

Analysis of social travel and social interaction patterns has become an important part of transportation research in recent years. Seminal studies in the transportation literature have documented theoretical frameworks and empirical evidence of how personal social networks influence travel choices (Carrasco and Miller 2006, Carrasco et al. 2008; van den Berg et al. (2009, 2010, 2013) ; Axhausen 2008). These studies addressed social networks and travel choices at a given time or age of an individual. However, social networks evolve (Snijders et al. 2010; Sharmeen et al. 2010) over time and life stages. Although the research frontier in activity-based modelling is shifting from cross-sectional models to short and long term dynamic models, research on social networks and travel has predominantly been concerned with analyses of static ego-centric social networks. Whereas, evidently, social networks and activity-travel patterns co-evolve (Sharmeen et al. 2014a). Ignoring such interdependency may lead to biases in prediction and erroneous design and output of models for policy evaluation. Thus, a challenge in travel behaviour research is to model the dynamics of social networks.

Attempts to formulate dynamic models of social networks can build on previous work on simulating social networks for population-wide predictions using the concept of tie formation (Arentze and Timmermans 2008; Arentze et al. 2012; Kowald et al. 2012; Arentze et al. 2013) and rational behavioural assumptions and network statistics (Hackney and Axhausen 2006; Hackney 2009; Hackney and Marchal 2011; Illenberger et al. 2009; Illenberger et al. 2012). Methods developed in these research can be used to predict tie formation. However, social ties may disappear over time, and the attributes of existing ties may also change over time. Hence, any model of the dynamics of social networks should take these different processes into account.

This paper suggest such a dynamic model. The aim of this study, in an attempt to contribute to the analysis and modelling of travel behaviour from a long-term perspective, is to formulate a model of the dynamics of social ties (i.e. tie retention or dissolution), in which lifecycle events are considered as triggers of change.

More specifically, the study is based on the utility-based tie formation model developed in Arentze et al. (2012, 2013). The model is extended to incorporate the influence of lifecycle events on social ties. The model draws from social network theories, the life course approach and takes geographical distance into account. The proposed model predicts the formation of and changes in social ties among individuals in the population. The empirical analysis demonstrates how the parameters can be estimated using a two-step likelihood estimation approach. We could not take the role of transitivity and reciprocity of decisions into account, owning to data limitation. However the model can be easily extended to incorporate those.

In the following sections, we will discuss the relevant theory and literature, methodology and approach, data and descriptive statistics, and the results of model estimation. We conclude the study by summarizing the major conclusions and delineating avenues of future research.

## Review of literature

There exists a long tradition of social network simulation in sociology, physics and health studies. In travel behaviour research, however, the formulation of simulation models is an emerging field of research. The focus of attention in most studies in travel behaviour research has been concerned with the prediction of activities and trips associated with mandatory and maintenance purposes. Discretionary activities and trips have received much less attention and have been modelled in rather minimalistic ways. Due to the increasing share of social and recreational activities and travel in societies all around the world, consideration of social travel in a more behavioural way is now generally seen to be essential. Therefore, a very relevant stream of research focuses on the ways social networks can be modelled for nationwide predictions.

The basic theories of social tie formation consider homophily, reciprocity and transitivity as core concepts. Homophily describes the notion that individuals’ preferences for social ties are based on the degree of similarity with others (McPherson et al. 2001). Reciprocity is a property often present in social networks and implies that social ties are two-sided, i.e. that if A is a friend of B then B is also a friend of A (Byrne 1971). Transitivity accounts for the existence of common friends; it describes that a tie between A and C is more probable if they have a common friend B (Bidart and Degenne 2005). Apart from that, accounting for geographical distance is also crucial since social networks of populations are distributed in geographical space. Studies provide evidence that social relationships are significantly influenced by geographical distances (Mok et al. 2010).

Hackney and colleagues (Hackney and Axhausen 2006; Hackney 2009; Hackney and Marchal 2011) were one of the firsts in transportation research to simulate social networks. They developed a multi-agent system using behavioural assumptions and network statistics. They incorporated the notion that new links may appear and existing links may disappear from an individual’s social network. The link removal algorithm uses a face-to-face social interaction parameter. If a link is not renewed by social interaction it can be removed after a threshold time has passed. Although this study does formulate a theory and model of social network dynamics, the results were not validated with empirical data. Illenberger et al. (2009, 2012) simulated social networks using a different approach, which is more focused on the spatial properties of social networks. They tested the model based on network indicators such as edge-length distribution and network degree distribution. On the downside the model they proposed does not take into account social network theories related to homophily and transitivity.

A comprehensive theoretical and modelling framework was developed by Arentze and Timmermans (2008) to predict the formation of social ties. A core assumption of this model is that the utility a person derives from social interaction is a function of dynamic social and information needs. The study is theoretical in nature and was not empirically tested. Arentze et al. (2012) proposed a link formation model and a method to simulate population-wide social networks. The model is consistent with the traditional social network theories (homophily, reciprocity) in the social science literature as well as with random-utility theory and takes geographical distance into account. The model has been estimated and applied in a case study using a dataset of ego-centric networks collected in the Netherlands (van den Berg 2012). It led to the conclusion that known properties of social networks can be reproduced by the model. The transitivity component was however not incorporated in this model. In a follow-up, Arentze et al. (2013) developed an extension of the model to incorporate transitivity. They estimated and tested the model using a Swiss dataset. The model considers relationships between persons that are relevant for leisure activities and provides insights on the connectedness between actors and the factors affecting the leisure relationships between them. Furthermore, the model is scalable to the populations generally involved in large-scale micro-simulation systems. Kowald et al. (2012) described an extension of the tie-formation model within this framework to take into account a wider set of person and location attributes.

All these studies considered social networks at a given point in time and therefore did not take the dynamics into account. The social network of a person may change as an individual grows older, changes home or job, etc. The decisions to form, retain or delete a tie is essentially a choice within the human behavioural paradigm. In other fields of research, different approaches of simulating the evolution of social networks have been explored (for an overview, see Doreian and Stokman 2013). Commonly used approaches include small world topology (Grabowski and Kosiński 2006), stochastic approximation algorithms (Snijders 2001), Bayesian hierarchical block models (Rodríguez 2012) and network equilibrium structures (Doreian and Stokman 2013). These models tend to focus on the structural distribution and properties of social networks and do not reflect any behavioural patterns of adaptation strategies. Snijders et al. (2010) using actor-based models incorporated behavioural patterns, but their approach has not been developed for population wide prediction. In summary, the lack of behavioural underpinnings and strong assumptions regarding network structure and topology limit these methodologies to be readily applicable to large scale simulation frameworks.

To that end, to predict changes in social networks, we propose and test a model of social network evolution, using lifecycle events as triggers of change. It is based on life course theory (O′Rand and Krecker 1990) to identify moments of change in an individual’s life. The lifecycle approach has previously been used to investigate changes in car ownership (Prillwitz et al. 2006; Oakil et al. 2014a), mode choice (Verhoeven et al. 2007; Oakil 2013; Oakil et al. 2011, 2014b) and activity and travel time allocation in general (Sharmeen et al. 2013a). It is argued that key events may induce stress and trigger individuals or households to make changes in their activity and travel schedules. Similarly, in this research we assume that lifecycle events may bring about changes in individuals’ and households’ experiences and also influence their relationships or ties (Elder Jr 1994; Elder 1998). Lifecycle events are therefore used to differentiate between two phases: an initial and an adaptation phase, referring to before and after event situations respectively. The model predicts new tie formation in both phases and modifications of existing ties in the adaptation phase. The purpose of the present study is to show how the model can be specified and estimated using empirical data. Application of the model to generate and simulate population-wide social networks is beyond the focus of the present study and left for future research. The next section explains the methodology in detail.

## Methodology and approach

The model is based on a utility-based tie formation function introduced by Arentze et al. (2012, 2013) . The model predicts the probability of the formation of a social tie between two persons in the population based on a random utility maximization approach. The utility is defined by three structural utility components related to respectively homophily, geographical distance and transitivity. Homophily refers to the phenomenon that individuals have a preference to form a social tie with other individuals who are similar to them. The influence of geographical distance is that, keeping everything else equal, the farther two persons are apart in geographical space the smaller the probability that there is a tie between them. Transitivity refers to the existence of common friends, which may facilitate the formation of a tie. As a general assumption of the model, friendship ties are reciprocal. In other words, if person *i* is a friend of person *j* then person *j* must also be a friend of person *i*. Of course, preferences may vary, but for the sake of simplicity at this stage, the model assumes that *U*_*ij*_ is equal to *U*_*ji*_. Formally, the utility of a tie is formulated as:

1 $$U_{ij} = V_{ij}^{Q} + V_{ij}^{D} + V_{ij}^{C} + \varepsilon_{ij}$$

where *U*_*ij*_ is the utility of forming a tie between individual *i* and individual *j* and $V_{ij}^{Q}$, $V_{ij}^{D}$ and $V_{ij}^{C}$ are structural utility components related to homophily, geographical distance and transitivity (common friends), respectively, and $\varepsilon_{ij}$ is an error term. The model therefore states that a tie between two individuals is more probable if the persons are similar in attributes, live nearby and have common friends. In this study, we leave the transitivity component out of consideration since we do not have relevant data and only look at the homophily and geographical distance effects on tie formation.

To account for the opportunity of meeting a person and/or the costs (time, money) associated with maintaining a tie, the model includes a threshold utility. The threshold values may differ between persons depending on the time they are willing or able to invest in maintaining social ties and possibly other constraints. A tie is worthwhile to make or maintain when the largest value of the threshold utility is met:

2 $$P\left( {i \leftrightarrow j} \right) = { \Pr }\left( {U_{ij} > \hbox{max} \left[ {u_{ij} ,u_{ji} } \right]} \right)$$

where *u*_*ij*_, *u*_*ji*_ are the threshold utility values for individual *i* and individual *j*.

**Fig. 1:**
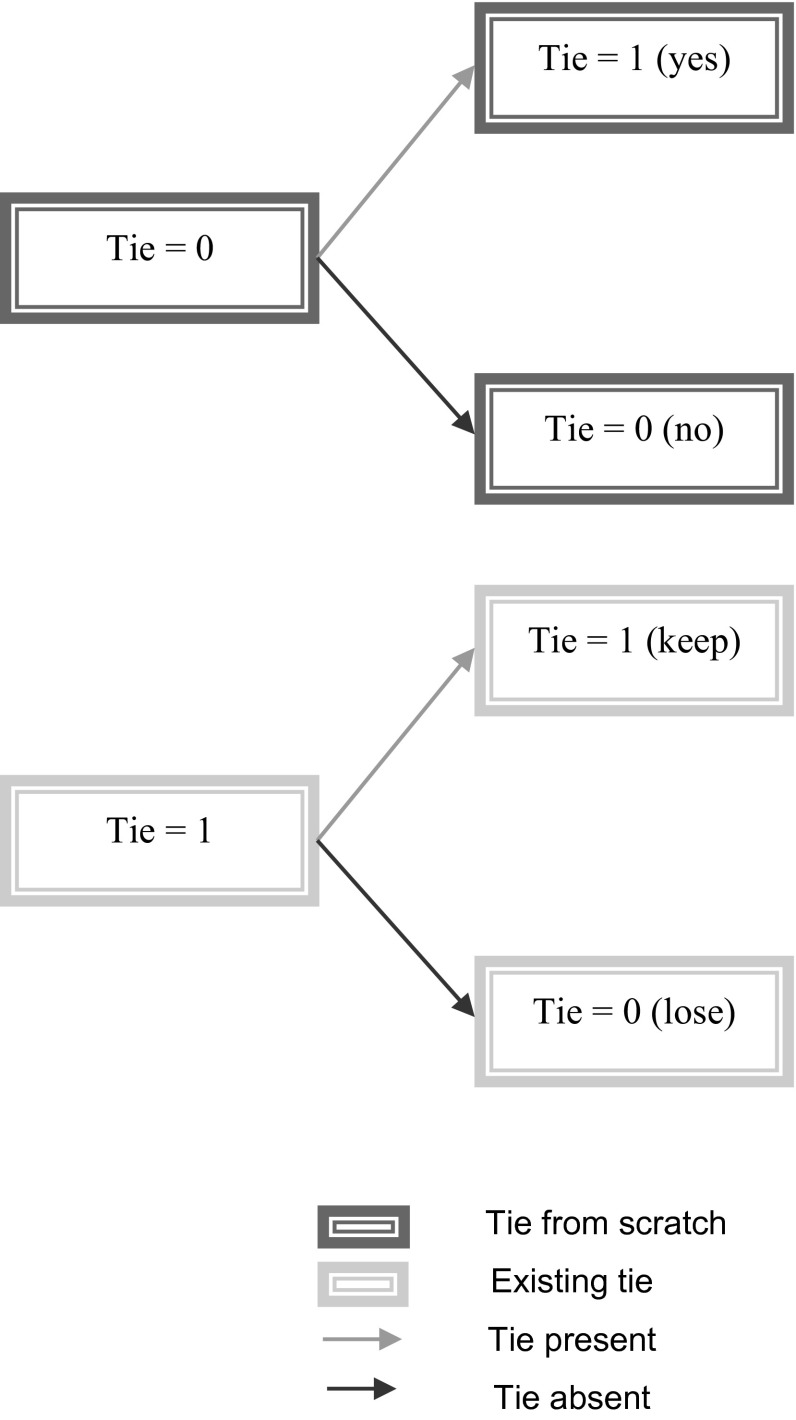
Problem definition (*top diagram*—no tie; choice is to make a tie or not, *bottom diagram*—tie exists; choice is to keep it or lose it)

To incorporate the dynamics induced by life cycle events in personal social networks, we model decisions of tie formation in two phases (before and after an event). In the initial phase individuals make tie-formation decisions as described by the basic model above. In the adaptation phase, an individual has two decisions to make (Fig. 1):

1. Whether to make a new tie or not
2. Whether to keep an existing tie or not (break/lose it)

The proposed extension of the utility function to cover both phases can be defined in operational terms as follows:

3 $$U_{ijg}^{{}} = \mu_{g} [V_{ijg}^{Q} + V_{ijg}^{D} + Z_{ig} ] + \alpha_{g} +_{i} \,+\, \varepsilon_{ij}$$

where *g* is an added index of existing condition, *α*_*g*_ is a condition specific constant, *η*_*i*_ is a random error component related to agent *i*, *Z*_*ig*_ is an additional term that captures the influence of the existing network and type of the event on the utility of a tie ($Z_{ig} = 0$ in the initial phase), *μ*_*g*_ is a condition-specific scaling factor and $\varepsilon_{ij}$ is a random error term as before. In the initial phase, *g* = 0 by definition and in the adaptation phase *g* = 1 if no tie exists and *g* = 2 otherwise. Thus, in the *g* = 2 case the function defines the utility of maintaining the tie and in the *g* = 0 and *g* = 1 case the utility of forming a new tie in the initial and adaptation phase respectively. In this equation, the constant, *α*_*g*_, captures the threshold utility of forming a new tie (*g* = 0, 1) or maintaining an existing tie (*g* = 2). The structural utility terms, *V*, on the right-hand side of the equations are operationalized for the different conditions as follows. First, in the initial phase where no tie exists (*g* = 0), the attribute-similarity utility is specified in a straightforward way as:

4a $$V_{ij0}^{Q} = \mathop \sum \limits_{k} \beta_{ik} X_{ijk}$$

where *X*_*ijk*_ is a homophily characteristic between person *i* and *j* regarding attribute *k* and *β* are parameters to be estimated. For the adaptation phase the term is extended to account for a possible re-evaluation of the same attributes in the adaptation phase, when the tie is new (*g* = 1) or already exists (*g* = 2):

4b $$V_{ij1}^{Q} = V_{ij2}^{Q} = \mathop \sum \limits_{k} \left( {\beta_{ik} X_{ijk} + \beta_{k}^{\Delta } X_{ijk} } \right)_{{}}$$

where $\beta^{\Delta }$ parameters represent adaptations of the evaluations. The distance related utility term is specified differently for the cases where the tie is new (*g* = 0 or *g* = 1) and already exists (*g* = 2), as follows:

5a $$V_{ij0}^{D} = \theta_{\text{i}} { \ln }(D_{ij} )$$

5b $$V_{ij1}^{D} = \theta_{\text{i}} { \ln }(D_{ij} ) + \theta^{\Delta } { \ln }(D_{ij} )$$

5c $$V_{ij2}^{D} = \theta_{\text{i}} { \ln }(D_{ij} ) + \theta^{\Delta } { \ln }(D_{ij} ) + \theta^{ + } (D_{ij}^{ + } ) + \theta^{ - } (D_{ij}^{ - } )$$

where, *D*_*ij*_ is geographical distance between persons *i* and *j*, $$D_{ij}^{ + }$$ is a dummy variable indicating an increase in distance for the tie caused by the event, $$D_{ij}^{ - }$$ is the a dummy variable indicating a decrease in distance and *θ*, $$\theta^{\Delta }$$$$\theta^{ + }$$ and $$\theta^{ - }$$ are related parameters. The log transformation of distance $$\left( {\ln D_{ij} } \right)$$ is implemented to take decreasing marginal utility of distance into account, which generally is assumed to be the case in tie formation models. Finally, the influence of the existing network and type of event is zero by definition in the initial phase (*g* = 0):

6a $$Z_{i0} = 0$$

and is defined for the adaptation phase as (if the tie is new or exists):

6b $$Z_{i1} = Z_{i2} = \mathop \sum \limits_{m} \tau_{m} E_{m} + N_{i}$$

where *E*_*m*_ is the *m*th dummy variable indicating the type of event, *N*_*i*_ is the size of the existing social network of person *i* and *τ* and *λ* are parameters to be estimated. To account for taste heterogeneity, the core parameters are included as random parameters in this model:

7 $$\beta_{ik} = \beta_{k} + \gamma_{ik}$$

8 $$\theta_{i} = \theta + \chi_{\text{i}}$$

where *γ*_*ik*_ and χ_i_ are agent-specific error terms regarding attribute similarity and distance parameters, respectively.

The parameters have the following interpretation: $\beta^{\Delta }$ parameters represent the effects of being in the adaptation phase on the way similarity is valued on the various attributes (*k*); *τ* parameters represent effects of particular events on a base utility (or threshold) of a relationship; *λ* represents the effect of the current size of the network in the adaptation phase on the utility of a relationship and *μ* takes into account a possible scale effect on how attributes and distance are valued under the different conditions. In the application a scale will be estimated for the condition where a relationship already exists (*μ*_2_) relative to the condition where the tie is new (*μ*_0_ = *μ*_1_ = 1).

In sum, the above Eqs. (3)–(8) define the model of tie formation decisions in the initial phase and adaptation phase taking into account the event that have taken place in the adaptation phase. The model takes into account the nature of the event as well as a possible change in geographical distance and allows for scale differences in the structural utility of a relationship depending on whether a relationship already exists or not, as well as for a difference in base utility (threshold) between these two conditions. Furthermore, the model takes into account taste-heterogeneity across agents (ego’s) in terms of homophily, geographical distance and base utility (threshold) for formation or keeping ties.

The model can be estimated using maximum likelihood estimation. For this estimation we need a dataset that contains a sample of social ties as well as a sample of non-existent social ties (Arentze et al. 2013). Furthermore, we need to have observations of tie dynamics (i.e. a tie that appears or disappears) and tie maintenance (ties that are retained) after a life cycle event. Finally, we need geographical distance changes (if any) for each pair of individuals after the event. In the next “Data preparation and descriptive anaylsis and Estimation results” sections we discuss the data collection and estimation method in more detail in the context of an application.

## Data preparation and descriptive anaylsis

**Fig. 2:**
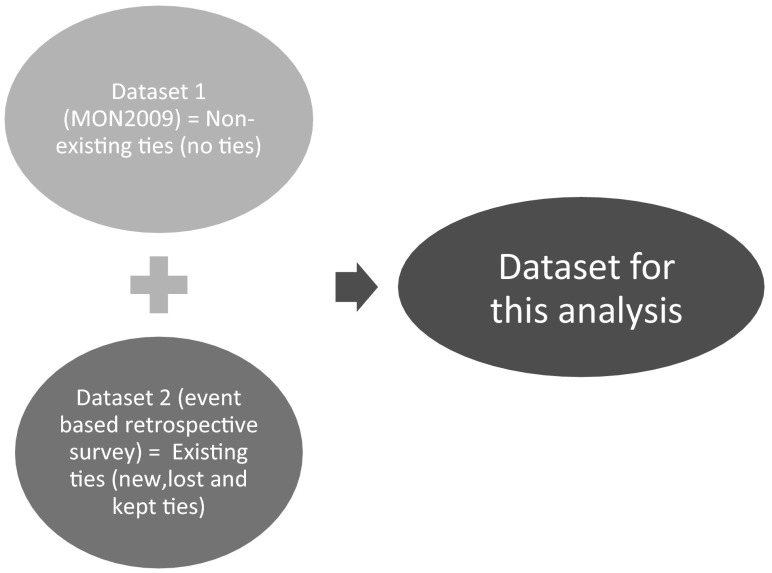
Schema representing which information was obtained from which dataset

Two datasets are used to estimate the model (Fig. 2). The first data set is from a national travel survey (MON) of The Netherlands collected by the Ministry of Transport (Ministry of Transport 2009). It is a travel-diary panel survey. For this study we have taken the latest version of 2009. It is a large sample (in 2009, approximately 30,000 individuals) representative of the Dutch population. The second dataset was collected to obtain information about the dynamics of personal social networks. A questionnaire was designed for an event-based retrospective survey where information was collected about changes in social networks of persons due to major life-cycle events (Sharmeen 2015). The first dataset is used to obtain a sample of the population. The sample is used to provide negative observations, i.e. the persons with whom no tie exists, for each individual. Although in principle all persons of the entire population with whom the person does not have a friendship relationship constitute negative observations, it has been shown that a sample suffices to obtain reliable estimates (Arentze et al. 2013) except for the constants^1^. The second dataset provides the key information to estimate a friendship model that allows us to predict probabilities of ego-alter tie dynamics.

A paper-based and web-based questionnaire survey was carried out in September 2011 in the Netherlands among a random sample of the population to collect data on social network dynamics. A criterion used to select respondents was that they must have experienced at least one life cycle event (viz. residential relocation, getting married/divorced/cohabitation, children starting school, starting new job and starting University) within the past 2 years. The following events were distinguished:

1. Residential relocation: change of residence.
2. Getting married/divorced/cohabitation: change in civil status.
3. Children starting school: a child in the household of the respondent started school for the first time.
4. Starting new job: the respondent started a new job that involves a change in the workplace.
5. Starting University: the respondent joined the University for higher education.

If multiple events have been experienced then the respondent was instructed to choose the most recent one to answer the survey questionnaire. If they satisfied this criterion, i.e. had experienced an event in recent years, respondents were forwarded to a set of questions related to the event. To better organize the study in terms of activity travel behaviour, the last two events were consolidated and named ‘change in job/study’ to indicate changes in mandatory activity and travel. The event of children starting school poses demands in maintenance activity and travel, whereas residential relocation can bring in changes in all types of activity and travel budget allocation. The event of change in civil status is a basic demographic event and was taken as a base event in the study.

The questionnaire was divided into four sections. Section one was about present socio-demographics, section two about details of tie changes due to life cycle events, section three about details of close social ties that were not affected by the life cycle events and section four about the changes in the weekly activity and travel schedule in response to life cycle events. The majority of the respondents were recruited by a survey organization having a dedicated panel, representative of the Dutch population. In addition to that a number of University students were sent invitations, using the list of newly admitted students at the Eindhoven University of Technology. Respondents were selected based on the question whether any of the stated events had occurred in recent years in their life. Only if the answer was affirmative, the respondent could proceed with the questionnaire.

In this study we use the data collected in sections one, two and three. In section one, in addition to the socio demographic characteristics, the respondents were asked to report the size of their present social networks according to type of relationships (family, friends, neighbours and other), excluding the members of own household. Section two and three are about detailed alter information for a subsection of personal social networks (changed ties and close ties respectively).

In section two, respondents were asked to report details of all ties that were changed (regardless to close ties or not, household members or not) as a result of the event. They were forwarded to a table where they had to list existing ties where a change occurred (if any) as well as new ties that were formed. For each listed tie where a change occurred they had to fill out the type of change (geographical distance, frequency of contact per mode both before and after the event), the socio-demographics of the alter and information about the tie (strength, length known). They also reported new ties and lost ties here. Lost ties mean that no social interaction has occurred anymore after the reported event. If no change in the composition of social networks were observed, they could indicate that and skip this section (for details of the questionnaire see Sharmeen 2015).

In section three, (concerning details about existing/retained personal social networks), respondents reported details about their close ties. Close ties were defined as those they share important information with, discuss personal problems, have regular contacts with or ask help during emergency or daily necessities. The details include age, gender, education level, geographical distance, strength, type and age of the relationship, and frequency of interaction (both face to face and via ICT). There was space for up to 25 ties.

Table 1 shows some descriptive statistics. Age was classified into five categories, viz. 0–19, 20–29, 30–39, 40–59 and 60+ years. In the Dutch education system secondary and tertiary education is divided into several levels that students can choose based on merit and personal goals (UNESCO-UNEVOC 2012). The education levels are categorized in six groups, viz. primary, general secondary, vocational secondary, post-secondary, undergraduate, graduate and higher. The sample is relatively homogenously distributed across education levels and age groups, with an exception of the (under represented) elderly group. Male are slightly over represented. The average number of working hours per week is 21.0 and the average number of children in the household is 1.0. The majority of the respondents have a driving license and the average size of the social network is 29.0.

**Table 1:** Sample descriptive of event-based retrospective survey data

Observed Variables	Description	Mean or Proportion (%)
Age ≤20	Age of ego is less than 20 years	20.2
Age 20–29	Age of ego is between 20 and 29 years	28.2
Age 30–39	Age of ego is between 30 and 39 years	24.9
Age 40–59	Age of ego is between 40 and 59 years	23.1
Age 60+	Age of ego is more than or equal to 60 years	3.5
Male	Ego is male	54.6
Primary	Highest education level is primary	8.4
General Secondary	Highest education level is secondary (general)	28.0
Vocational Secondary	Highest education level is secondary (vocational)	18.7
Post-secondary	Highest education level is post-secondary	22.0
Undergraduate	Highest education level is undergraduate	11.7
Graduate and higher	Highest education level is graduate or higher	11.2
# workhr	Number of working hours per week	21.60
# child	Number of child in the household	0.97
Driving License	Having car in the household and the ego has driving license	79.6
Social Network Size	Size of close social network	29.02
Event: Res relocation	Ego changed home location in past 2 years	31.1
Event: Civil status	Ego started living together or got married or separated or divorced in past 2 years	14.2
Event: Child start sch	Children of the household (under 18) started school	23.0
Event: Job/Study	Ego started university for higher education/New job	31.7
# Lost ties	Number of ties lost after the event	1.44
# New ties	Number of new ties after the event	1.62

**Fig. 3:**
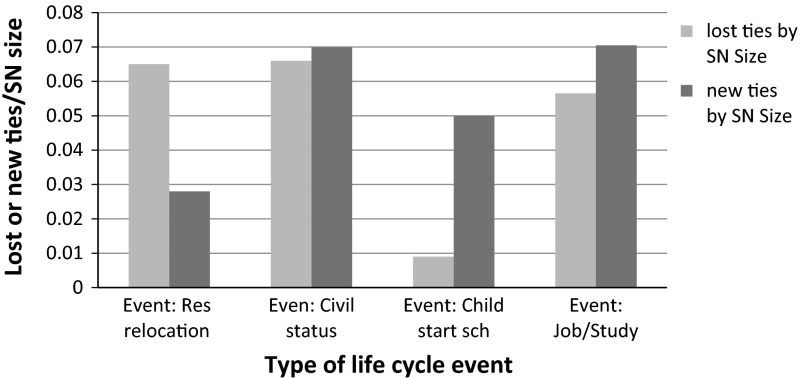
Social network dynamics according to life cycle events

The average number of ties lost is 1.44 and average number of new ties is 1.62 per respondent (Table 1). The proportion of ties lost per type of life cycle event is shown in Fig. 3. The differences between types of life cycle events are remarkable. Residential relocation has a larger impact on lost ties than new ones. The opposite is true for the event of children starting school. Change in civil status (defined by a change in cohabitation, wedding, separation and divorce) has a more equal impact in terms of lost and new ties. The proportions are among the highest compared to other types of life-cycle events. The proportion of new ties is the highest for the event of new job or study (Fig. 3).

**Table 2:** Overview of sample of ties in initial and adaptation phases

Phases	Type of ties	# of ties
Initial (before event)	Before 0 after 0 (no)	82,100
	Before 0 after 1 (yes)	8021
Adaptation (after event)	Before 0 after 0 (no)	887
	Before 0 after 1 (new)	326
	Before 1 after 0 (lost)	68
	Before 1 after 1 (kept)	827

As stated above, a sample was drawn from the national travel survey data to account for negative ties existing in the population^2^. The sample accounted for 0.56 % of the Dutch population in the Netherlands. From the retrospective survey data ties reported for the before-event case (collected in section three of the questionnaire plus the lost ties reported in section two) are relevant for the initial (before event) phase. In total 8,021 ties were reported (positive observations) and 82,100 persons represented the negative observations (i.e. persons in the population with which no tie existed) in this phase (Table 2).

The new ties that were made and the ties that were lost after the event are relevant data for the adaptation phase. Since the numbers of new ties are very small compared to the big population-wide sample and the number of lost ties is very small compared to the number of maintained ties, we applied sampling techniques to prepare the data for the adaptation phase. First, for the decision to form new ties, a random sample of 887 was drawn from the population-wide sample. Second, for the decision to maintain existing ties, a random sample of 827 ties were drawn from the 8021 existing ties (Table 2). By using the sampling method the parameters can be estimated except for the constants (*α*) in the utility functions which will be biased.

Table 3 presents the distributions of ties in the initial phase based on the similarity between the socio demographic characteristics of ego and alter. The columns present the percentage distribution of ties present and absent in terms of the homophily attributes and geographical distance between the ego and the alter.

**Table 3:** Data descriptive for initial (before event) phase (90121 cases)

Ego-alter socio demographics comparison	Tie present (%)	Tie absent (%)
Same age	46.7	13.7
Age difference by 1 category	28.7	27.5
Age difference by 2 categories	11.1	23.4
Age difference by 3 or more categories	13.5	35.4
Different age (total)	53.3	86.3
Same gender	62.8	49.8
Different gender	37.2	50.2
Same education level	12.6	9.2
Education level difference of 1 category	33.6	16.4
Education level difference of 2 categories	16.8	14.3
Education level difference of 3 or more categories	37.0	60.1
Different education level (total)	87.4	90.8
Distance (mean) in km	38.4	133.207

For each category, column totals are equal to 100 %, for instance same gender and different gender for ‘tie present’ column the total equals 100 %. The same holds for ‘tie absent’column

We can observe strong homophily effects for gender and age, and almost no homophily effects for education level. Approximately 87 % of the ties were formed with individuals having different education levels. This is somewhat different than what was reported by Kowald et al. (2012) using a Swiss dataset. They reported homophily effects for both age and education level (in their study presence of tie was 14 % higher if ego and alter had same education level than different education levels). However, it is to note that the categories used in the Swiss dataset are broad (mandatory school, apprenticeship and university) and not directly comparable to the Dutch system. The age categories are rather comparable. Furthermore, it is to note that the name generators used in our survey picked up a significant number of family ties and siblings which are more likely to be dissimilar in education levels but some may fall in the same age category (such as, siblings and cousins). Nonetheless the difference in the statistics with respect to education level is remarkable.

## Estimation results

Using a mixed-logit framework, the model was estimated based on the total set of 92,229 observations (Table 2) with 200 Halton draws. Biogeme 2.0 was used to estimate the model. A mixed logit formulation was used to account for the panel-structure of the data, i.e. multiple observations for the egos of the sample. The estimation was conducted in two steps. At first the scale parameter, μ (in Eq. 3), was estimated in a multinomial specification of the model. Then the data were rescaled accordingly and the model was estimated using the final mixed logit estimation with the scale parameter omitted (given that the data have been rescaled and hence the scale difference is solved). This two-step procedure was used since existing software packages for estimation do not allow scale parameter estimation in a mixed-logit framework. The best fitting model was obtained after evaluating random effect variations in constants, same age group and distance parameters (Table 4). The log likelihood estimates and Rho square statistics display that the model has a good fit compared to a null model.

**Table 4:** Binary mixed logit model of social ties formation and maintenance (population wide prediction)

Parameter estimates	$\beta$	t-stat
A. Initial (before event) phase		
Constant	−2.63	−21.16
Same age group	1.46	23.73
Age difference by 2 category	−0.538	−8.43
Age difference by 3 categories	−0.998	−10.17
Same gender	0.541	14.41
Same education level	−0.0365	−1.4
Education level difference by 2 categories	−0.264	−4.24
Education level difference by 3 or more categories	−0.961	−8.48
Log of distance in km	−3.38	−43.52
B. Adaptation (after event) phase	Effects on *β*	t-stat
Constant	−2.77	−3.58
Same age group	−0.715	−2.99
Age difference by 2 category	0.369	0.67
Age difference by 3 categories	1.23	2.32
Same gender	−0.182	−0.63
Same education level	1.23	3.36
Education level difference by 2 categories	0.275	0.69
Education level difference by 3 or more categories	1.04	2.74
Log of distance in km	3.24	13.77
Event: Relocation with other events	1.26	2.65
Event: Change in work/study hr	0.732	2.86
Event: Children of the household starts school	1.34	2.93
Size of social network (close ties)	−0.0339	−0.12
C. Existing ties (with a history)		t-stat
Constant	3.49	6.08
Scale	1.55	3.95
Decrease in geographical distance	6.64	7.41
Increase in geographical distance	−1.03	−1.46
Std Dev Random Effects	$\eta_{i}$, $\gamma_{ik}$ and $\chi$	t-stat
Constant	1.86	23.13
Same age group	1.18	14.48
Distance	0.622	7.1

Dependent variable: tie present (kept for existing ties) or absent (lost for existing ties)

*Model goodness-of-fit information:* Initial log-likelihood: −63935.896; Final log-likelihood: −15373.5; Rho-square: 0.759; # Halton draws: 200, # observations: 92229

The model constants are negative for both initial and adaptation phases, which is expected, indicating all else being equal the probability is that a tie is not made.

Strong homophily effects are indicated by the parameter estimated for the initial phase. Results indicate that the probability of making a tie with an individual of same age group is higher. The utility of a tie decreases monotonically for the age differences. This is a plausible finding; it reflects that friendships among the same age group are more probable (Kowald et al. 2012). Similar findings are exhibited for actors with same gender.

Same education level has no significant effect on the utility of a tie in the initial phase estimate. However, difference in education level of two categories does have a negative effect on the utility and this negative utility further increases when the difference further increases (to three or more categories). This implies that making friends with someone with a relatively large difference in education level is relatively improbable which coheres to the homophily effect. The findings related to homophily are comparable to the similar Swiss study conducted by Kowald et al. (2012). However, in the latter study a positive effect of same education was found in addition to the negative effects of education differences of two or more categories.

Distance^3^ has a negative effect on the utility of a tie, which is expected and in line with the general notion of the effects of proximity. As the geographical distance between two individuals increases, the probability of a tie between them decreases. Thus geographical distance still matters in formation of ties despite the advancement of information and communication technologies, as was also reported in earlier studies (Mok et al. 2010)

Utility effects for the adaptation phase (after a lifecycle event) are estimated as effects to the base (initial/before life cycle event phase) parameters (Eq. 4b). The effects of age variables are opposite in sign to those at the initial phase; this suggests that in the adaptation phase, the size of homophily effects is reduced (note that the parameters should be interpreted as effects on the parameters of part A). Specifically the correction parameter is high for the largest age difference category (age difference by three categories). The effects are also reducing the effects for same education level and education level difference by three or more categories, indicating a decrease in homophily tendency and even a marginal inclination towards heterophily (for three or more categories difference in age and education level) in the adaptation phase. The adaptation phase has no significant effect on the utility of same gender. The effect of distance however is strongly reduced in the adaptation phase. To summarize, the estimates of the effects of the adaptation phase suggest that when the social network is existent, an individual is less sensitive to similarity in age, education and geographical proximity compared to the initial phase where a network is being formed.

In terms of individual specific variables, such as type of events and size of social network, findings suggest that the utility of forming a new tie or maintaining an existing tie increases for all three types of events when compared to the base event type (i.e. change in civil status). The utility effect is largest for the event of a child in the household starting school and the lowest for starting a new job or study. All these events potentially create opportunities of meeting new people as new activity and/or travel spaces are introduced, either in the form of a new neighbourhood, school for children, job or education place. The size of the existing social network, however, does not have a significant effect on the utility of a tie. Evidently the social network size does not matter in making new friends or maintaining existing ones. Note that by definition the existing social network here is limited to the close friends.

For existing ties, we calculated a scale parameter to correct for a possible difference in scale of utility when the tie exists and the choice is about keeping the tie or not. The scale parameter is positive (1.55) indicating an increase of the effects of structural homophily and distance variables when a tie exists. Therefore, for existing ties, the effects are strengthened. In other words, both the decrease in homophily and an inclination towards heterophily is intensified.

The heterophily effects can be explained as a major part of the ties that are retained after the event are family ties, which tend to include an age difference and may include gender difference. Investigation related to social interaction dynamics confer that individuals keep in touch with family ties in one way or another. If face to face interaction frequency has been reduced after an event, it was compensated by increased amount of contacts using ICT (Sharmeen and Timmermans 2011; Sharmeen et al. 2013b).

Finally, in terms of geographical distance findings suggest that a decrease in geographical distance increases the utility of keeping the tie. An increase in geographical distance has a negative sign but the effect is not significant. In our previous empirical study (Sharmeen et al. 2014b), we found evidence that changes in geographical distance and local accessibility indicators affect maintenance of existing ties. Nonetheless we can emphasize that changes in geographical distance are important and should be taken into consideration in determining maintenance of existing ties.

## Conclusion

The study offers a method to estimate a model to predict the evolution of social networks, triggered by life cycle events. Incorporation of social networks into dynamic activity-travel demand models requires a methodology to predict the probabilities of social tie formation, change and disappearance. Existing research in this regard is limited to begin with, being a relatively new frontier in transportation. Recent promising progresses (e.g. Arentze et al. 2012; Kowald et al. 2012 and Arentze et al. 2013) have been made to predict and simulate static social networks. The contribution of this paper is extending the method towards dynamic analysis and prediction (therefore not only considering friendship formation but also friendship maintenance). The proposed model predicts the formation of and changes in social ties among two actors in the population. The empirical analysis demonstrates how the model can be estimated.

The relevance of this model is appealing not only to researchers but also to practical analysts and policy evaluators. With respect to travel demand forecasting the need to simulate and predict transitions of individuals in well recognized (Ben-Akiva and Bowman 1998; Bhat and Koppelman 1999; Timmermans et al. 2010; Timmermans et al. 2014). On the other hand, social network generates and influences activity and travel patterns (Carrasco and Habib 2009). Therefore, we need similar approach to simulate the dynamics of social networks. Otherwise, by definition, the input to dynamic and quasi static models of travel demand forecasting will be biased. Hence to improve the application of activity-based models of travel demand, a model to simulate the dynamics of egocentric social networks, in which it is assumed that changes in social networks are triggered by lifecycle events, is formulated and estimated. For policy formulation, a method to include social network dynamics in the forecasting and policy analysis framework is needed. In addition, the prediction of social network dynamics through life cycle events can provide useful insights to practitioners and policy makers not only in transportation but also in the fields of health, psychology and even crime prevention.

The model derived from previous work is based on theories of social networks, such as homophily, reciprocity and degree distribution. To determine the social ties between two individuals in the population it uses the random utility maximization concept. The utility of a tie is measured by the degree of similarity between the actors and the geographical distance between them. A tie between two individuals is worthwhile only if the utility exceeds a threshold value for both persons involved. Thus the model predicts if a new tie emerges or not between two individuals. Further, the model is extended to also predict behaviour in an adaptation stage in case a life cycle event occurred.

Findings suggest that the formation of new ties is influenced by homophily between the actors in the initial phase. The effects however are small in the adaptation phase, in the sense that sensitivity to homophily declines or in some cases a marginal inclination towards heterophily can be observed. For existing ties the effects become stronger; both decrease in homophily and an inclination towards heterophily is intensified. A major part of the ties that are retained after the event are family ties, which may explain the heterophily effects. This finding is plausible since family ties are not maintained by choice, like other types of ties. Separate analysis for different types of ties could provide more detailed insight, which I suggested for future research.

In addition to that, geographical proximity influences the formation of a tie. With respect to existing ties we find that a decrease in distance due to some event has a positive effect on tie maintenance. In line with an earlier study (Sharmeen et al. 2014b), these findings suggests that not only prevailing but also variations in geographical distance affect friendship formation and maintenance.

The model can be used to simulate dynamics of social networks for a population wide distribution. Demographic projections are the basis of forecasting methodologies and scenario analysis frameworks in any field, to which this study contributes. Using the model one can create a social network at the initial phase and then update friendship relationships in response to particular life cycle events. It can be used for scenario analyses regarding particular changes in population demographics with life cycle events. Further, studies have argued that present behaviour and choice decisions are not independent of past choice heuristics. Yet, in static models of social networks the effects of path dependencies are often disregarded. Using this model the changes in social networks can be predicted taking path dependency effects into account.

The model estimation is based on a selected sample of individuals who experienced a lifecycle event in the past 2 years. There might be some inconsistencies in data, since the sample drawn from the population were not screened for life cycle events within past 2 years. The sampling was done purposefully to report the effects of long term lifecycle events on social networks, a trait that is completely absent in the literature. However, this sampling poses hindrance in using the model for predicting changes not triggered by lifecycle events. A comprehensive longitudinal data collection is needed to also capture autonomous changes. As a first empirical finding of predicting social tie dynamics the research shows promising results.

In addition, there are a number of limitations and possibilities of extending the model. First, data limitations have prevented us from incorporating effects of reciprocity and transitivity (having a common friend). Tie formation and deletion decisions are reciprocal. Those decisions depend on both ego and alter and can be affected by lifecycle events experienced by both parties. In the present model only one sided decisions were considered. The influence of having common friends (or circle of friends) on tie formation/deletion was also not captured in the present study. Second, due to budget limitations some important lifecycle events, such as childbirth, were not included in the survey and remains as future work. Furthermore, other geographic attributes such as density, accessibility and urban form can be incorporated to extend the model to cater for the variety of geographical places. Moreover work status and mobility patterns of the actors can determine the possibilities to make new ties, to some extent. For instance, a full time worker and a long distance commuter may have different possibilities of meeting new people than a part time student or a full time homemaker. For larger and mixed communities, cultural differences should also be taken into consideration when applying this model across populations.

## Footnotes

1 The use of sampling for estimation is a well-known method in discrete choice modelling. Sampling leads to biases in the estimation of constants; methods exist to correct for such biases (King and Zeng 2001) which is relevant when the model is to be used for prediction.

2 In two years’ time attributes of the cohorts may change due to lifecycle changes. To take such changes into account one would need to have longitudinal panel data which was not available in the present study.

3 Distance was taken as a continuous variable in this study. Introducing categories, such as, walking distance, within the same postcode/neighbourhood/village/city could add some meaningful information and is worth mentioning. We did not do this because of two reasons. First categorisation would essentially mean losing some information. Second, we aimed for a model that could be generalized across the population. Definition of walking distance may vary for each individual. Similarly, districts, cities and postcode areas vary in size and in the provision of infrastructure facilities and therefore are not comparable.
